# The economic burden of functional neurological disorder

**DOI:** 10.1098/rstb.2024.0470

**Published:** 2026-09-17

**Authors:** Siddhartha Bandyopadhyay, Joanne L Baker, Jisan Afrin

**Affiliations:** University of Birmingham, Economics, Birmingham B17 0SE, England

**Keywords:** FND, economics, cost-effectiveness

## Abstract

We provide an analysis of the economic burden of functional neurological disorder (FND). For a condition that is complex to diagnose and treat, estimating its true prevalence and incidence can be challenging. This estimation challenge has economic implications in terms of directing healthcare and other resources to mitigate the burden of FND. Existing economic analysis is limited, with most studies ignoring the wider social costs of FND, which are believed to be considerably higher than direct healthcare costs. This, in turn, affects the reliability of cost-effectiveness analysis. Using a number of existing studies, we provide an analysis of some of the challenges in estimating the true costs of FND. We revisit the debate around the appropriateness of cost-effectiveness analysis using quality-adjusted life years (QALYs) versus other frameworks, particularly generalised risk-adjusted cost-effectiveness (GRACE), in diseases like FND and its equity implications. Finally, we discuss how the probable underestimation of the economic burden of FND stifles innovation and dampens investment in promising technology.

This article is part of the discussion meeting issue ‘Digital healthcare for the management of functional neurological disorders’.

## 1. Purpose and background

The purpose of this opinion piece is to understand the challenges of estimating the economic burden of functional neurological disorder (FND), provide a critical overview of current estimates of this burden, discuss its limitations and suggest ways to improve estimates that better reflect patients’ willingness to pay. In doing so, we outline a proposed framework that may be used to do a cost-effectiveness analysis that is more responsive to the preferences of patients. Finally, we discuss the implications of underestimating the costs of FND in terms of (dis)incentivizing investment and innovation that may improve the well-being of patients with FND.

FND is complex to diagnose and treat. The National Institute of Neurological Disorders and Stroke (NINDS^1^ ) defines FND as ‘a neurological condition caused by changes in how brain networks work, rather than changes in the structure of the brain itself, as seen in many other neurological disorders. Physical symptoms of FND are genuine but cannot be explained by changes in the brain structure.’ The NINDS website states that FND symptoms may include: seizure-like episodes and movement problems, as well as problems with cognitive function. Other symptoms include dizziness, speech difficulties, problems with vision and hearing, numbness or inability to sense touch.

Their origin arises primarily from a disorder of the nervous system functioning [1]. Stone *et al.* [1] further point out that ‘…many clinicians have had little formal clinical education on the assessment and management of these disorders, and patients are often not offered potentially effective treatments.’ This means that the incidence and prevalence of FND are probably underestimated. Further, the estimates in the published literature on incidence and prevalence have wide variation. A review by Finkelstein *et al.* [2] confirms this: they find that the incidence of FND (based on 19 studies that report it) was estimated at 10–22/100 000, while minimum prevalence of FND was estimated at 80–140/100 000, with a possible range of 50–1600/100 000 (based on 21 studies that report it). These are wide confidence intervals that arise from different methodologies used in the study, particularly the type of FND reported, with e.g. some studies reporting all categories of FND while others report on some important sub-types, such as functional seizure. The sources from which they reported cases also affect the recorded prevalence and/or incidence owing to the differing expertise across these medical settings, e.g. whether they are from primary care units or more specialized units where there are higher chances of being diagnosed. The review authors focus on low-bias studies, which are those on functional seizures. They do data extrapolation to get the best estimates, which they still believe to be underestimates. In addition to the complexity of diagnosis, socio-economic and cultural factors may contribute to FND not being diagnosed, including the stigma associated with the condition.

These wide intervals around incidence and prevalence pose a problem in estimating the economic burden of FND, as any estimates of the total economic burden would also have considerable variation, which thereby makes decisions on resource allocation challenging. However, it is not in just the incidence or prevalence that we see variation; there is also variability in per-patient cost across studies that affects the computation of total excess burden. The purpose of this piece, as outlined earlier, is to clarify conceptual issues on measuring costs and, more importantly, on the appropriateness of using the standard quality-adjusted life-years (QALYs) framework (which we discuss in the next section) to measure patient benefits, consider alternatives and their impact on innovation.

Thus, we do not undertake a systematic review, and in fact, although not plentiful, two recent reviews do exist as we discuss below. Nonetheless, we have done a brief search on PubMed Central and google scholar on the economic impact of FND using search terms such as costs of FND, economic burden of FND, cost-effectiveness of treatments, etc., to get an understanding of the range of estimates and to identify any recent studies that are not covered in the reviews. These searches were independently conducted by all authors, who made judgements on papers that were most relevant, and also searched for systematic reviews on the economic costs of FND. The systematic reviews were examined for relevant papers and compared to the results of our search. It was reassuring that all the papers we read to enhance our understanding were included in the reviews, except for one that was published after the most recent review. We only refer to some of the relevant papers to make our points around where the variability in economic burden stems from.

It is useful to clarify that by economic burden, we mean both the direct medical costs and the indirect costs borne by the individual and society at large. Many of the papers we found focus on direct medical costs, but like other complex conditions such as post traumatic stress disorder (PTSD), where the indirect costs (e.g. loss of productivity, unemployment and social care costs) are higher [3], this also appears to be true for FND, as some estimates suggest. There are some exceptions that do measure some societal costs (e.g. productivity loss), even if as a secondary analysis (e.g. [4–7]). However, systematic reviews of the economic costs are few and far between.

Indeed, there are only a few evidence reviews that examine the economic costs of FND [8,9]. These studies suggest a considerable cost of FND, but cost-effective interventions may not have been adequately identified, possibly because the particular interventions were not cost-effective or owing to the study's limitations. There can be a number of reasons for this, not least if the wider social costs of FND have been ignored or underestimated, as is often the case. In addition, if FND is present with other comorbidities then this may make the excess burden of FND both conceptually and empirically hard to quantify. Further, as mentioned above, FND cases are widely underestimated, and hence estimates of total excess burden would be lower than the true excess burden. This also has implications for cost-effectiveness analysis if there are economies of scale and a secondary but possibly bigger dynamic impact, because skewed cost-effectiveness estimates can also affect the market for innovation in FND treatment.

The paper by O'Mahony *et al.* [8] is the only detailed analysis of economic evaluation apart from a more recent shorter report [10]. Further, these focus mainly on clinical/psychological intervention and do not analyse the cost-effectiveness of technological inventions.

A complementary set of interventions look at technology to aid FND patients. We have consulted papers that do look at cost implications for technological innovations, mainly VR and robotics. Many of the studies show variability around how economic viability (broadly speaking) is measured, and the details provided make it difficult to judge the precise economic efficacy of the intervention. Our judgement on these papers appears to be borne out by the synthesis of the evidence from two surveys that do look at technological interventions. One of these is a systematic review by Cano-de-la-Cuerda *et al.* [11] and another by Agbemanyole *et al.* [12], with both reviews suggesting that many of the papers use very loose definitions of cost-effectiveness.

## 2. Conceptual issues around measuring cost-effectiveness

Here, we provide more detailed thoughts on some conceptual issues around measuring the cost of FND that we alluded to above and principles that might help us understand what the benefits of different treatments (including technological interventions) could be and the challenges that lie with measuring such benefits. The first is the variability in FND types and heterogeneity in response. This leads to wide variations in estimates that we have seen, depending on how the sample is constructed (e.g. from GPs versus neurological units) as well as the types of health outcomes included. As mentioned, if the wider social costs of FND dwarf the direct health costs, this affects not only the total excess burden but also the calculations on the cost-effectiveness of treatments, which probably explains why the reviews are unable to locate cost-effective interventions. To take an example, Nelson-Sice *et al.* [6] did a cross-sectional study of FND patients in the UK attending tertiary neurology and neuropsychiatry clinics, reporting that the mean direct healthcare cost was £3328 per patient, with inpatient care accounting for nearly 59 percent of total costs. Indirect costs were substantially higher, with employment loss (£6594) and informal care (£9255) exceeding direct NHS expenditure. Second, there can be considerable social impacts that are difficult to measure, e.g. the impact on friends and family, including intergenerational ones. Partnering directly with patients and their carers to co-design research may help us identify some of these issues, but quantitative measurement remains a serious practical issue.

In this context, it is always useful to draw on evidence from a similar disease, and one can, of course, look at other, more well-defined neurological conditions. However, given its link with trauma [13] and its complexity, examining the costs of PTSD could be useful as there may be similarities in some of the estimation challenges and hidden costs. Thus, while they are different conditions, recent work on estimating the challenges of PTSD provides useful insights into the challenges around estimating the excess burden of PTSD. Both have shared risk factors, such as trauma and adverse childhood experiences. Indeed, as reported in the theoretical background of the guidance ([13], p. 19), the following is instructive: ‘Historically, FND was often seen as a post-traumatic condition linked to sexual abuse and/or other forms of childhood maltreatment. FND is sometimes treated in adults using an adapted post-traumatic stress disorder (PTSD) model (e.g. [14])’. While current understanding does not conflate the two, it recognizes that those with FND have a much higher probability of also experiencing PTSD owing to the same underlying trauma than the general population. Thus, differences in the two conditions notwithstanding, referring to a recent paper that calculated the excess cost of PTSD provides a sensible benchmark for what these estimates might look like. It also provides clues on the type of hidden costs that we are probably missing.

A third issue that can affect treatments’ cost-effectiveness is the choice of control group (or even its explicit presence in some studies). Even if a randomized controlled trial (RCT) is done, the ‘treatment as usual’ is not easy to identify and may involve differing standards of treatment/care. To demonstrate this, the following examples show how radically the cost-effectiveness of different treatments can vary. Goldstein *et al.* [4] conducted a large, multicentre RCT (the CODES trial) comparing cognitive behavioural therapy (CBT) plus standardized medical care with standardized medical care alone for adults with dissociative (non-epileptic) seizures. The accompanying economic evaluation was until recently considered the only comprehensive cost-effectiveness study of an FND intervention. It reported that CBT improved psychosocial functioning and treatment satisfaction but produced an incremental gain of only 0.0152 QALYs, resulting in an incremental cost-effectiveness ratio (ICER) of £120 658 per QALY, exceeding the NICE cost-effectiveness threshold (at that time £20 000–£30 000 per QALY; [15] which has since been updated to £25,000–£35 000 per QALY , see https://www.nice.org.uk/what-nice-does/faqs/changes-to-nice-s-cost-effectiveness-thresholds). The lack of cost-effectiveness holds even when social costs are accounted for, but the standard medical care model (the control group) is too intensive for most FND treatment, thereby affecting the cost-effectiveness analysis. Since then the Physio4FMD study [16] has been reported on which is another multi centre RCT that reports a mean incremental cost per QALY of £4,133 without taking into account health and social care costs. It also analyses the wider societal costs associated with FND and concludes that physiotherapy is cost-effective compared with treatment as usual with a very high probability particularly when these wider societal costs are taken into account.

Smaller UK pilot studies have explored the cost-effectiveness of specific therapeutic interventions. Nielsenet *et al*. [17] conducted a randomized feasibility trial of physiotherapy for patients with functional motor disorder, reporting a mean incremental cost per QALY gained of £12 087. Reuber *et al.* [18] piloted a brief psychodynamic intervention for patients with mixed functional neurological symptoms; assuming that quality-of-life gains were maintained for one year, the mean incremental cost per QALY gained was £5328. Both studies demonstrated potential economic efficiency compared with resource-intensive psychological therapies, although their small sample sizes and limited follow-up restrict generalisability (as summarized in [8]). However, the lack of a control group makes it difficult to undertake a proper cost-effectiveness analysis.

Finally, the variability of FND diagnosis and uncertainty about appropriate approaches across cases create challenges in the market for innovation. Investors may be wary of putting up money to fund innovation, and an underestimate of incidence and prevalence also affects the amount that governments invest in either directly funding studies on new innovations or in subsidizing new treatments.

In the next section, we look at whether the use of QALYs as a way to capture the benefits of an intervention is appropriate. This briefly considers the criticisms of QALYs, as well as particular issues that come up when applied to FND patients.

## 3. Revisiting the debate on QALYs for cost-effectiveness analysis and comparison with GRACE

QALYs are a widely accepted way of measuring health outcomes that incorporates both quantity and quality of life and hence can be easily compared across health conditions and interpreted easily. It is the most common summary outcome measure of the value and benefit of health outcomes^2^. This is used to quantify the effectiveness of a particular intervention and inform health policy, which is useful because the benefits of different interventions are multi-dimensional. QALYs combine the impact of gains in quality of life and quantity of life (i.e. life expectancy) associated with an intervention. The incremental QALYs (i.e. differences between two or more alternatives) compared with the incremental costs provide the measure of their economic value. Hence, this is widely used in cost-effectiveness analysis comparing incremental benefits against costs of different types of interventions.

QALYs are based on valuations of health-related quality of life measured on a scale where full health is valued as 1 and death as 0, with less than full health being given a score based on answers to a standard health questionnaire, and these valuations/scores are termed utilities. For example, if someone experiences a health state with a utility of 0.8 for 10 years and then a health state with a utility of 0.5 for 5 years (and then dies), their aggregate QALYs will be (0.8 × 10) + (0.5 × 5) = 10.5 QALYs. A detailed critique of QALYs is beyond the scope of this article, but the interested reader may wish to read Rand *et al.* [19] and Rand and Kesselheim [20], who summarize these critiques. They conclude that QALYs are still the most effective means for assessing cost-effectiveness in Health Technology Assessment (HTA). Two points critiquing QALYs are worth noting in this context; QALYs are considered to be both insensitive to health states as well as not incorporating non-health-related outcomes. Thus, QALYs make assumptions about patient preferences that are at odds with how economists generally quantify preferences over goods and services. Unlike standard structure on preferences that economists are used to dealing with, where utility is dependent on the current quantity (e.g. you may incrementally value a good less once you consume a certain number of units), a unit QALY gain is considered to be the same whether one is moderately or severely ill. The non-health-related outcomes are also not usually accounted for in cost-effectiveness assessment, which has direct implications for diseases like FND, where direct healthcare costs are much lower than wider social costs. Thus, the non-inclusion of costs beyond direct healthcare ones can be a serious issue, although in principle cost-effectiveness using QALYs can include such non-healthcare costs, and as we saw above, there are studies that do so. But although they can be incorporated in principle, if they are not included in decisions involving policy and practice or used as a secondary outcome, interventions that help with non-health-related costs would be consistently undervalued. We discuss below an alternative to using QALYs for cost-effectiveness given the rise of cost-effectiveness analysis frameworks that are more responsive to preferences.

In terms of being more responsive to patient preferences and, in particular, conditioning willingness to pay on the health state of the patient, Lakdawalla and Phelps propose the generalized risk-adjusted cost-effectiveness (GRACE) framework and its generalization (e.g. [21–23], also [24]), which also compares this with other proposed frameworks such as equal value of life-years gained (EVL) and healthy years in total (HYT). In simple terms, GRACE does the following: it starts with the current health state of the patient (‘how sick am I?’) and asks how much they would be willing to pay for an improvement in their health condition. They answer that it is plausible that sicker patients will have a higher willingness to pay for the same incremental increase in utility, so someone with a health state of 0.3 will pay more to move to a health state of 0.4 than someone would to move from 0.9 to perfect health. Further, they argue that it is plausible that sicker patients would be more willing to give up their longevity (quantity of life) to get a better quality of life. They argued persuasively at a Royal Society meeting in 2024 that these apply well to diseases like FND, where symptoms can be severe and long-lasting. Consequently, these patients may be willing to take greater risks than those in good health, making the assumption that willingness to pay is independent of health state highly problematic.

A framework that does not take these considerations into account can also have unintended consequences, leading to a public and/or political backlash against the use of cost-effectiveness in healthcare allocation. Thus, the problems with QALYs in cost effectiveness analysis (and hence availability of treatment options) have in fact led to recent debates where, in the USA, there has been a direct ban on its use since the House of Representatives passed the Protecting Health Care for All Patients Act (HR 485) that prohibits its use in coverage and payment determinations under federal healthcare programmes—a move that has been lamented by a group of economists [25]. In a response to this piece, Lakdawalla and Doctor [24] acknowledge that the lack of an economic basis would be damaging for optimizing healthcare, but they make a compelling case for GRACE as a more equitable and patient-centric way of applying such cost-effectiveness.

While it may seem improbable that the UK will abandon an economic basis for funding healthcare in general (nor are we in any way arguing that they should), more precision in when it departs from QALY in cost-effectiveness is helpful. Our argument is that these issues are important in and of themselves (i.e. it is appropriate that patient preferences are well reflected and healthcare allocation is equitable), but this also has important derived effects on the market for innovation. For example, if the use of QALYs for cost-effectiveness directly discriminates against patients with FND then it also affects the market for innovation in this area because innovations that will be funded based on QALY gain may lead to a secondary effect, because it dampens the market for innovations that would have been considered cost-effective if patient preferences were taken into account. It might apparently seem that GRACE is less suitable in a centralized system of healthcare decision-making, such as in the UK, where decision-making based on QALYs is fairly entrenched. However, even in the UK, there are always exceptions made in allowing certain treatments that would not meet the effectiveness threshold under traditional QALY gains for serious conditions like cancer. One hopes that as understanding about FND increases, there will be some exceptions in what is funded there as well. But rather than make exceptions in an ad hoc way, the use of a well-accepted framework such as GRACE for measuring benefit to the patient would be far preferable, as that will be more equitable as well as provide more predictability that would incentivize the market for innovation.

## 4. Conclusion and implications for practice and policy

FND is a serious and complex health issue. What is known so far suggests that it is underdiagnosed, and the estimations of its economic costs are considerably uncertain owing to (i) substantial variation in incidence and prevalence estimates, as well as (ii) incomplete measurement of indirect and societal costs. Further, there may be methodological problems in evaluating the cost-effectiveness of treatments through the standard QALY framework. In conclusion, we summarize some of these below, with their implications.

The complexity of FND diagnosis means that potentially helpful treatment is often not offered, or offered with a delay. This delay, in turn, has a considerable economic impact as it may lead to healthcare costs that could have been avoided. FND also has considerable costs beyond those stemming from direct healthcare utilization. These wider societal costs are estimated to be far higher in magnitude than direct healthcare costs, which implies that cost-effectiveness analysis of treatment is significantly affected if only direct medical costs are considered. This is compounded by considerable variation in the estimates of incidence and prevalence, which makes it hard to obtain precise estimates of the economic burden of FND. Studies that look at cost-effectiveness vary in quality, and the lack of reliable estimates of incidence and problems of attributing the costs to FND (owing to the presence of co-morbidities) also affects cost-effectiveness analysis. Further, when trying to understand the cost-effectiveness of interventions, technological, therapeutic or otherwise, one needs to be sure that the comparison group is appropriately chosen to ensure the precise measurement of treatment impact. One also needs to explicitly consider the costs of inaction, e.g. increased hospitalization or use of A&E, in addition to the disutility of patients owing to diagnosis delay, as well as the wider societal costs that misdiagnosis or delayed diagnosis can cause.

Consideration of patient preference is particularly challenging if there is heterogeneity in response at different stages of the disease and for different individuals, with a patient's willingness to pay as well as their acceptance of risk depending on their current health state. In this instance, this has important implications for the suitability of the use of standard measures such as QALYs for cost-effectiveness, which may be particularly problematic because willingness to pay is not dependent on the current health state. Thus, alternative measures such as GRACE should be considered, as this would make spending on FND more equitable and responsive to patient preferences. There is an urgent need to have more granular studies that can quantify the impact of FND. This has implications not only for better targeting of resources and treatment, but also for incentivizing innovative activity that can lower direct and indirect costs associated with FND. This dynamic aspect is just as important as the market for innovation, and it is affected by the scale of the problem and how the cost-effectiveness of newer treatment options will be measured. This has important implications for the incentives for the private sector to invest in the development of technology that may reduce healthcare costs as well as improve independence of patients (and hence reduce societal costs). It will also affect the allocation of public funding (e.g. R&D grants). Thus, better understanding and diagnosis of FND should be complemented by more accurate measurement of its wider societal burden, in the hope of achieving cost-effectiveness by using an equitable path that is more responsive to patient preferences. This will both improve support of patients using existing treatment options and encourage more investment in R&D across the public and private sectors.

Thus, this paper draws attention to several conceptual and measurement issues in the economic evaluation of FND, an under-researched neurological condition, which has important implications for health policy and resource allocation more generally, including how the market for innovation is affected as a result.

## Data Availability

This article has no additional data.

## Footnotes

^1^

https://www.ninds.nih.gov/health-information/disorders/functional-neurologic-disorder; an offi cial website of the US government.

^2^
The definition and description are partly taken from https://www.yhec.co.uk/glossary-term/quality-adjusted-life-year-qaly/.

## Theme

One contribution of 12 to a discussion meeting issue ‘Digital healthcare for the management of functional neurological disorders’.
